# Supplementary material for: Challenges in the discovery of tumor-specific alternative splicing-derived cell-surface antigens in glioma
Source: Sci Rep. 2024 Mar 16;14:6362. doi: 10.1038/s41598-024-56684-0 (PMC10944514; doi:10.1038/s41598-024-56684-0)
Supplement: Supplementary file 2 — Supplementary Information. [file 41598_2024_56684_MOESM2_ESM.pdf]

Supplementary Information

Challenges in the discovery of tumor-specific alternative splicing-derived cell-surface antigens in glioma

Takahide Nejo [1], Lin Wang [1], Kevin K. Leung [2], Albert Wang [1], Senthilnath Lakshmanachetty [1], Marco Gallus [1], Darwin W. Kwok [1], Chibo Hong [1], Lee H. Chen [1], Diego A. Carrera [1], Michael Y. Zhang [1], Nicholas O. Stevers [1], Gabriella C. Maldonado [1], Akane Yamamichi [1], Payal B. Watchmaker [1], Akul Naik [3], Anny Shai [1], Joanna J. Phillips [1,4,5], Susan M. Chang [1,4], Arun P. Wiita [3,4,6,7,8], James A. Wells [2,4,9], Joseph F. Costello [1,4], Aaron A. Diaz [1,4], Hideho Okada [1,4,8*]

[1] Department of Neurological Surgery, University of California, San Francisco, CA, USA. [2] Department of Pharmaceutical Chemistry, University of California, San Francisco, CA, USA. [3] Department of Laboratory Medicine, University of California, San Francisco, CA, USA. [4] Helen Diller Family Comprehensive Cancer Center, University of California, San Francisco, CA, USA. [5] Department of Pathology, University of California, San Francisco, CA, USA. [6] Department of Bioengineering and Therapeutic Sciences, University of California, San Francisco, CA, USA. [7] Chan Zuckerberg Biohub, CA, USA. [8] The Parker Institute for Cancer Immunotherapy, CA, USA. [9] Department of Cellular and Molecular Pharmacology, University of California, San Francisco, CA, USA.

* Correspondence: hideho.okada@ucsf.edu

Supplementary Tables

Table S1. TCGA sample list analyzed in this study.

Table S2. GTEx sample list analyzed in this study.

Table S3. Positive sample rate data in intron retention analysis.

Table S4. Exon-exon junctions detected at each filtering step.

Table S5. Positive sample rate data of exon-exon junctions in tumor (TCGA) and normal (GTEx) tissues.

Table S6. Molecular pathology information of spatially mapped clinical tumor dataset.

Table S7. Molecular pathology information of longitudinally collected clinical tumor dataset.

Supplementary Figures

Fig. S1. Distribution of gene expression TPM values of the eight genes in the TCGA and the GTEx datasets.

Fig. S2. Supportive and corresponding wild-type junction read counts of the identified 13 AS events and *EGFR*vIII in TCGA and GTEx data.

Fig. S3. Positive sample rate of the identified 13 AS events and *EGFR*vIII in GTEx normal tissue samples.

Fig. S4. External dataset validation of the identified 13 AS events.

Fig. S5. Uncropped image of the agarose gel.

Fig. S6. Top 50 isoforms identified in the full-length transcript amplicon sequencing.

Fig. S7. Relative signal intensity distributions of wildtype proteins corresponding to the candidate AS events.

Fig. S8. Distributions of RNA-seq total read counts and detected junction counts between the TCGA and the GTEx datasets.

Supplementary Figure S1

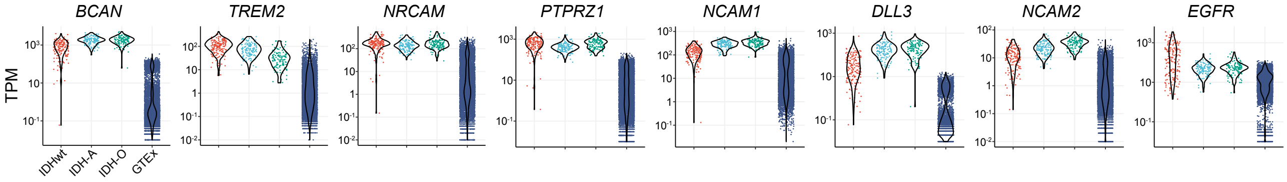

	average TPM	t-test *P* (v. GTEx)	average TPM	t-test *P* (v. GTEx)	average TPM	t-test *P* (v. GTEx)	average TPM	t-test *P* (v. GTEx)	average TPM	t-test *P* (v. GTEx)	average TPM	t-test *P* (v. GTEx)	average TPM	t-test *P* (v. GTEx)	average TPM	t-test *P* (v. GTEx)
all TCGA	1504.9	1.5×10^{-122}	82.1	1.1×10^{-85}	168.8	9.3×10^{-132}	588.5	7.2×10^{-120}	249.1	1.0×10^{-120}	144.4	3.5×10^{-61}	22.6	9.5×10^{-99}	184.7	1.5×10^{-17}
IDHwt	901.0	8.7×10^{-39}	115.5	3.7×10^{-47}	181.5	2.3×10^{-51}	676.0	6.5×10^{-47}	140.0	2.6×10^{-38}	50.1	4.9×10^{-15}	13.0	7.7×10^{-30}	386.9	3.5×10^{-14}
IDH-A	1878.4	8.4×10^{-56}	82.7	1.5×10^{-36}	147.1	2.5×10^{-47}	428.9	4.2×10^{-48}	292.1	2.6×10^{-64}	199.6	1.7×10^{-27}	22.9	3.9×10^{-45}	53.4	5.2×10^{-23}
IDH-O	1894.1	7.1×10^{-49}	36.8	1.4×10^{-19}	176.1	1.2×10^{-39}	651.5	2.4×10^{-41}	347.0	2.8×10^{-55}	208.6	4.7×10^{-32}	35.3	1.7×10^{-49}	62.5	1.8×10^{-18}
GTEx	13.3	-	4.3	-	16.3	-	13.4	-	32.6	-	0.7	-	2.4	-	16.0	-

Supplementary Fig. S1 Distribution of gene expression TPM values of the eight genes in the TCGA and the GTEx datasets.

Dot and violin plots depicting the gene-level expression of the eight genes that generate the pass-filter candidate AS events in each sample group, identical to **Fig. 2b**. Average TPM values for all TCGA-glioma, IDHwt, IDH-A, IDH-O, and GTEx groups are displayed in the bottom table, along with corresponding P-values calculated using a t-test against the GTEx samples.

Supplementary Figure S2

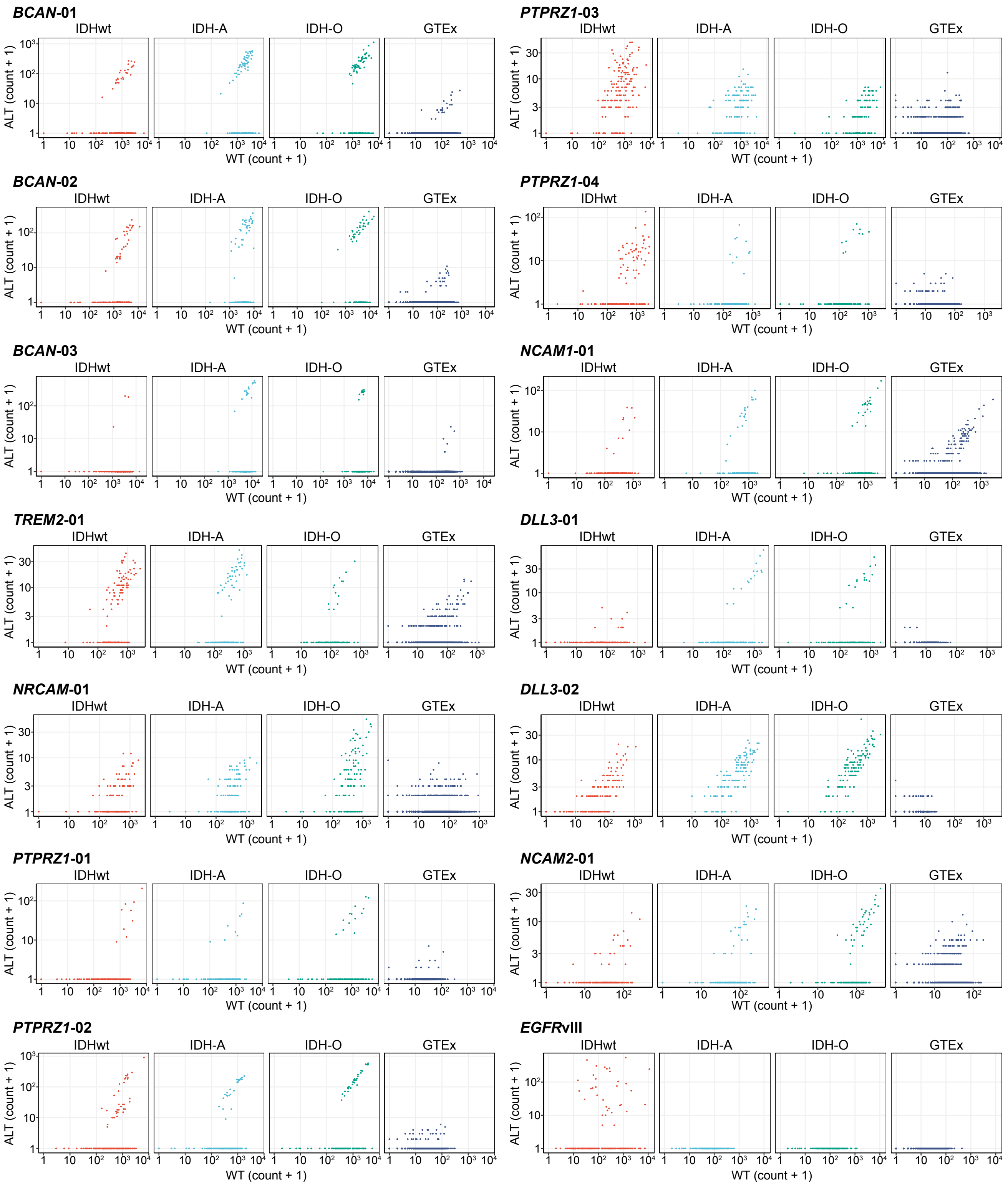

Supplementary Fig. S2 Supportive and corresponding wild-type junction read counts of the identified 13 AS events and *EGFR*vIII in TCGA and GTEx data.

Scatter plots illustrating the supportive and corresponding wild-type junction read counts of the identified 13 AS events and *EGFR*vIII in GTEx normal tissue samples, corresponding to **Fig. 2c–p**. For each event, the x-axis represents the corresponding wild-type (WT) junction read counts, and the y-axis represents the event supportive (ALT) junction read counts, respectively.

Supplementary Figure S3

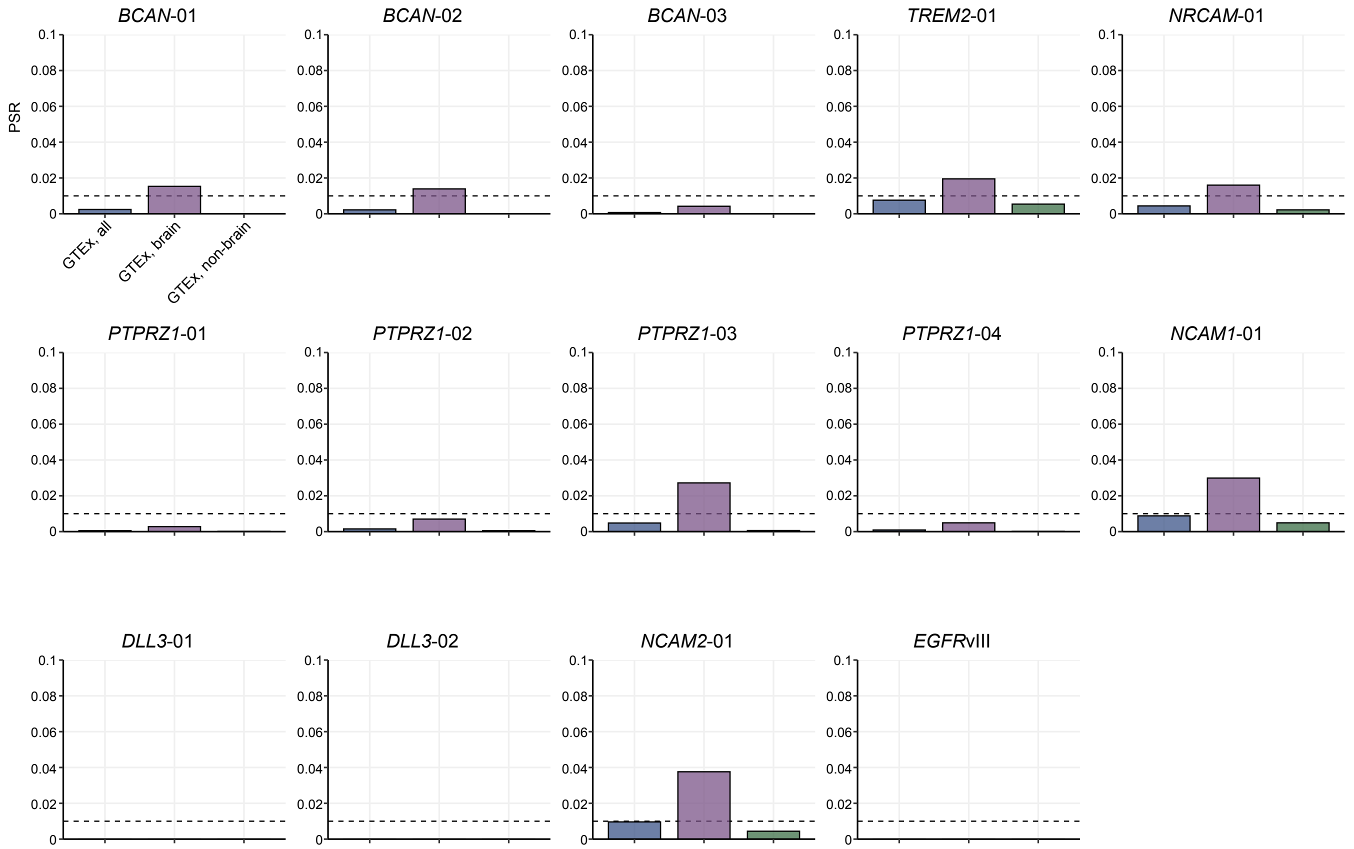

Supplementary Fig. S3 Positive sample rate of the identified 13 AS events and *EGFR*vIII in GTEx normal tissue samples.

Bar plots depicting the positive sample rate (PSR) of the identified 13 AS events and *EGFR*vIII in GTEx normal tissue samples, corresponding to **Fig. 2c–p**. The data are presented for all (n = 9,166), brain (n = 1,436), and non-brain tissue samples (n = 7,730). Break lines in the figures represent PSR = 0.01 (1%).

Supplementary Figure S4

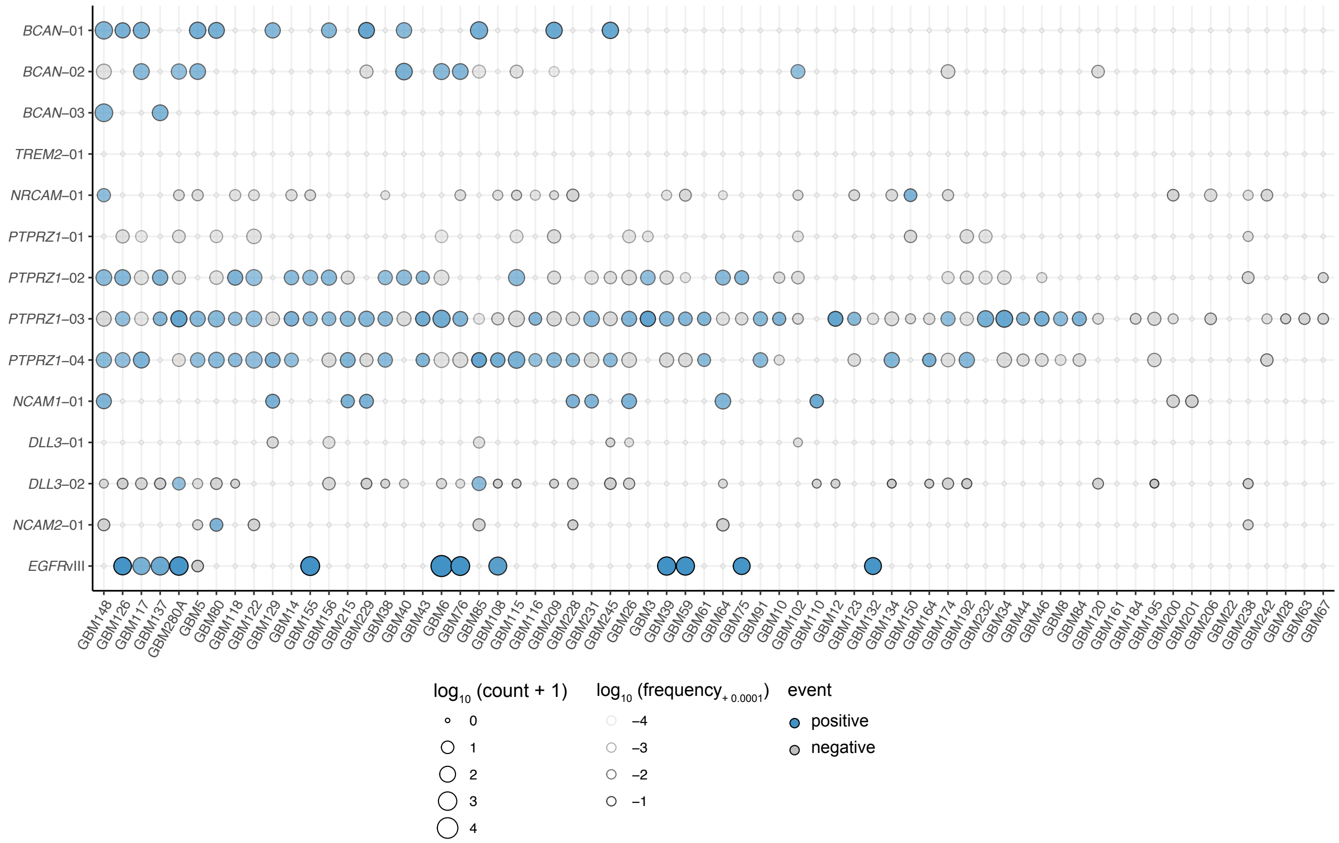

Supplementary Fig. S4 External dataset validation of the identified 13 AS events.

Dot plots displaying presence of candidate AS events in the Mayo PDX-GBM transcriptome dataset (n = 66 samples). Blue dots indicate events defined as positive, while gray dots indicate those defined as negative in each sample. The size and color intensity of each dot represent the positive read counts and positive read frequencies, respectively.

Supplementary Figure S5

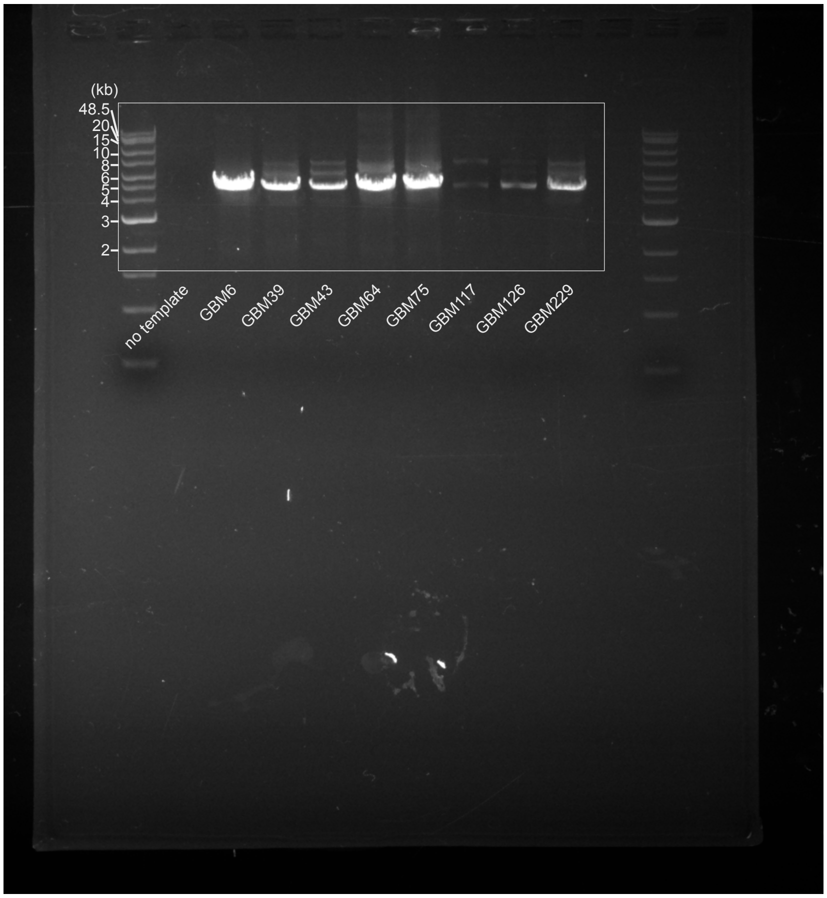

Supplementary Fig. S5 Uncropped image of the agarose gel.

Uncropped image of the agarose gel corresponding to **Fig. 3b**.

Supplementary Figure S6

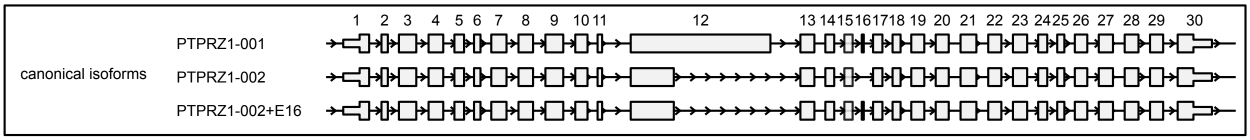

color	rank	template	ES/IR	neojunctions
	1.	PTPRZ1-002+E16		
	2.	PTPRZ1-002		
	3.	PTPRZ1-002+E16		+ AS-02
	4.	PTPRZ1-002+E16		+ AS-01
	5.	PTPRZ1-002		+ AS-02
	6.	PTPRZ1-002		+ AS-01
	7.	PTPRZ1-002+E16		+ AS-01/02
	8.	PTPRZ1-002+E16	+ ES-E2	
	9.	PTPRZ1-002+E16	+ IR-I7	
	10.	PTPRZ1-002		+ AS-01/02
	11.	PTPRZ1-001		
	12.	PTPRZ1-002+E16	+ ES-E11	
	13.	PTPRZ1-002+E16	+ ES-E6	
	14.	PTPRZ1-001		+ AS-04
	15.	PTPRZ1-002+E16	+ ES-E2	+ AS-02
	16.	PTPRZ1-002+E16	+ ES-E11	+ AS-02
	17.	PTPRZ1-002+E16	+ ES-E5	
	18.	PTPRZ1-002	+ ES-E2	
	19.	PTPRZ1-002+E16	+ ES-E2	+ AS-01
	20.	PTPRZ1-002+E16	+ ES-E11	+ AS-01
	21.	PTPRZ1-002	+ ES-E15	
	22.	PTPRZ1-002+E16	+ ES-E29	
	23.	PTPRZ1-002	+ ES-E11	
	24.	PTPRZ1-001		+ AS-02
	25.	PTPRZ1-002	+ ES-E6	
	26.	PTPRZ1-001	+ cryptic intron	
	27.	PTPRZ1-002+E16	+ ES-E27	
	28.	PTPRZ1-002	+ ES-E28	
	29.	PTPRZ1-002	+ ES-E14	
	30.	PTPRZ1-002+E16	+ ES-E28	+ AS-02
	31.	PTPRZ1-001		+ AS-01/02/03
	32.	PTPRZ1-001		+ AS-01
	33.	PTPRZ1-002+E16	+ ES-E19	+ AS-02
	34.	PTPRZ1-001		+ AS-02/04
	35.	PTPRZ1-002	+ ES-E19	
	36.	PTPRZ1-002+E16	+ ES-E3	
	37.	PTPRZ1-001	+ ES-E16	
	38.	PTPRZ1-002	+ ES-E11	+ AS-02
	39.	PTPRZ1-002+E16	+ ES-E28	+ AS-01
	40.	PTPRZ1-001	+ ES-E16	+ AS-01

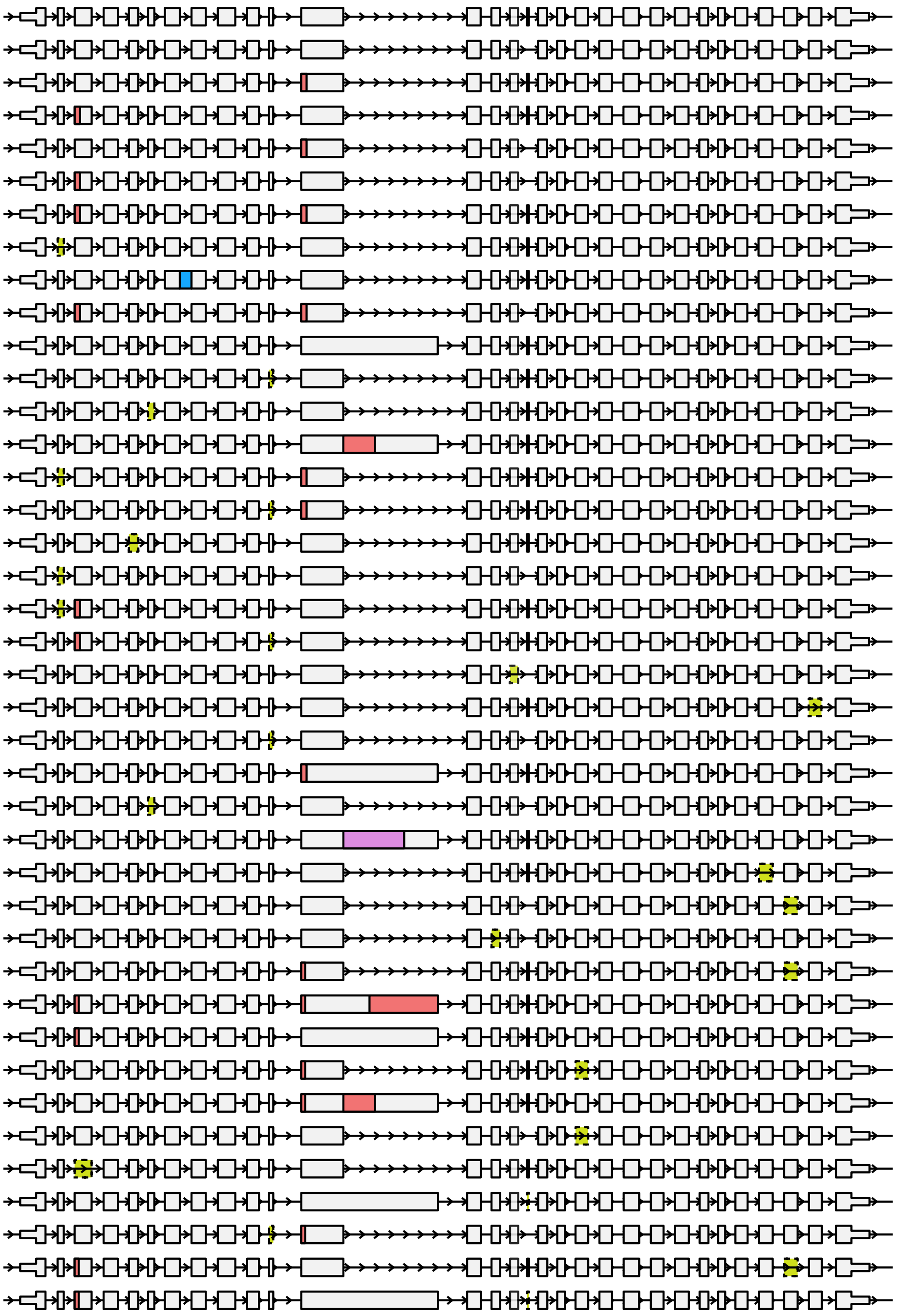

Supplementary Figure S6 (continued)

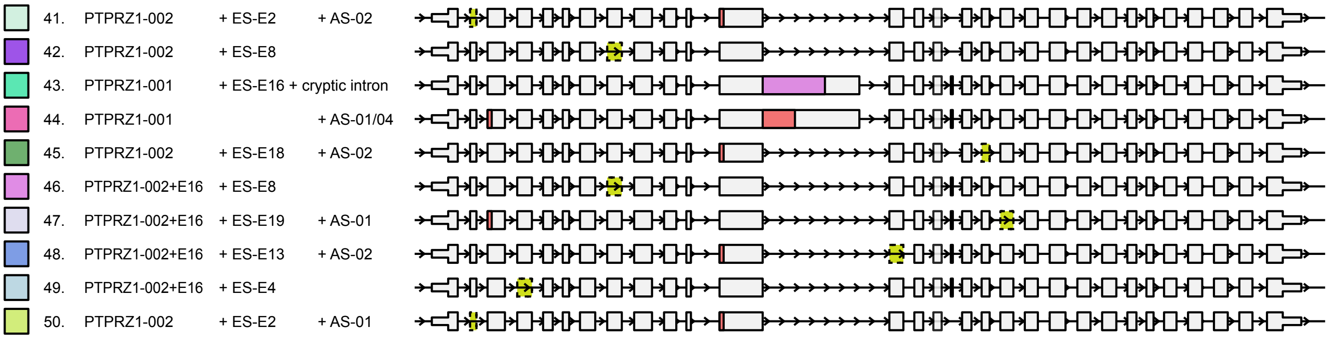

Supplementary Fig. S6 Top 50 isoforms identified in the full-length transcript amplicon sequencing.

Schematic illustration of the top 50 most frequently identified isoforms including canonical and cryptic. The colors in the left square correspond to **Fig. 3e**. The rank number indicates the ranking in the list. The four AS patterns identified through short-read sequencing (PTPRZ1-01, -02, -03, and -04) are highlighted in red and labeled as AS-01, -02, -03, and -04, respectively. Additionally, other events, such as exon skipping (yellow), intron retention (blue), and cryptic intron (purple) events, are also indicated.

Supplementary Figure S7

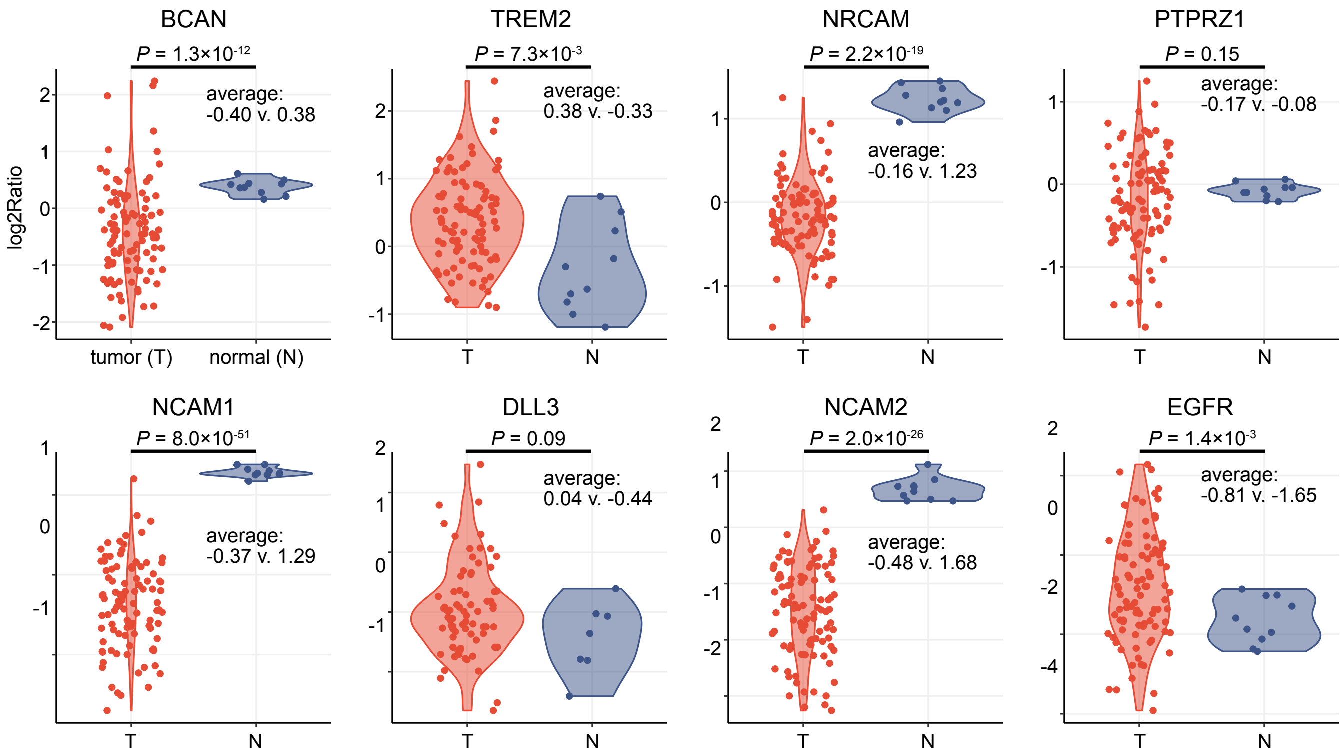

Supplementary Fig. S7 Relative signal intensity distributions of wild-type proteins corresponding to the candidate AS events.

Dot and violin plots showing the distributions of relative signal intensities for each of the wild-type proteins corresponding to the candidate AS events. The data has been sourced from the CPTAC-GBM data portal and is presented in its raw form, without additional modifications or transformations. The Y-axis represents log2 ratios to internal control reference samples.

Supplementary Figure S8

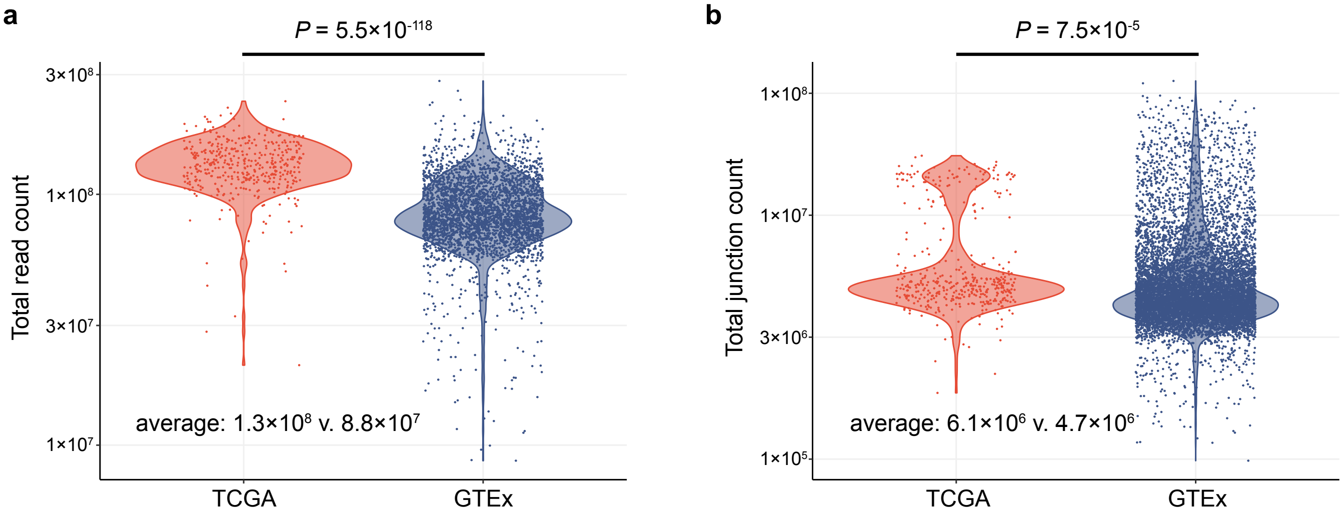

Supplementary Fig. S8 Distributions of RNA-seq total read counts and detected junction counts between the TCGA and the GTEx datasets.

Dot and violin plots showing the distributions of total read counts within the RNA-seq BAM file (**a**) and the total unique junction counts within the SJ.out.tab file (**b**) of each sample. *P* values are calculated using *t*-test.
